# Neurosyphilis: Discussing the Diagnostic Challenges

**DOI:** 10.1002/ccr3.70504

**Published:** 2025-05-09

**Authors:** Samuel Amo‐Tachie

**Affiliations:** ^1^ University of Ghana Medical School, University of Ghana, Korle‐Bu Accra Ghana; ^2^ Mamprobi Hospital Accra Ghana

**Keywords:** neurosyphilis, nontreponemal tests, penicillin, treponemal tests

## Abstract

Neurosyphilis can be difficult to diagnose without a high index of clinical suspicion. The diagnostic process itself may be challenging, especially when there is heavy reliance on CSF findings. Empirical therapy with benzathine penicillin after positive blood serology combined with suggestive clinical findings could be lifesaving for patients.

## 1 Introduction

Syphilis, thanks to the advent of antibiotics, has an uncomplicated course compared to ancient methods. A dose of “regular” benzathine penicillin is enough for a cure in most cases. Again, no matter the complexity or degree of advancement of the disease, the simplicity of the treatment does not change much, as penicillin is widely available [1]. The challenge comes when it advances to the central nervous system (neurosyphilis) or cardiovascular system (cardiovascular syphilis), and there is not much but clinical findings to point clinicians to the diagnosis. In other words, it is more challenging to diagnose advanced syphilis than to treat it, even though some damages are irreversible [2]. There are various manifestations associated with neurosyphilis depending on which parts of the central nervous system get involved, such as the meninges, cerebral cortex, etc. [3].

## 2 Case History/Examination

A 54‐year‐old man with no significant medical history presented with a week's history of incoherent speech and an inability to maintain balance while walking. There was no antecedent history of similar occurrences and no associated fever, neck stiffness, vertigo, or tinnitus. Further history revealed bladder incontinence since the onset of the symptoms. He was not an alcoholic, nor did he have a history of the use of recreational drugs; he had about four lifetime sexual partners, but the previous three were all before he married his wife of about 25 years. There was no recall of genital sores in the past; his blood pressure and all other vital signs on arrival were unremarkable.

His physical examination showed an ataxic gait with a tendency to fall toward his left side and a positive Romberg sign. He also had impaired proprioception in both lower limbs, alongside mild weakness on his left side. His pupils were about 3 mm and reacted equally to light. He was not oriented to time and place on arrival; Kernig and Brudzinski signs were negative; he did not have dysdiadochokinesia, but deep tendon reflexes were diminished. All other examination findings were normal.

### 2.1 Differential Diagnosis

With these findings, differentials included stroke, cerebellitis, demyelinating disorders, normal pressure hydrocephalus, vitamin B12 deficiency, and neurosyphilis.

### 2.2 Outcome and Follow‐Up

**FIGURE 1:**
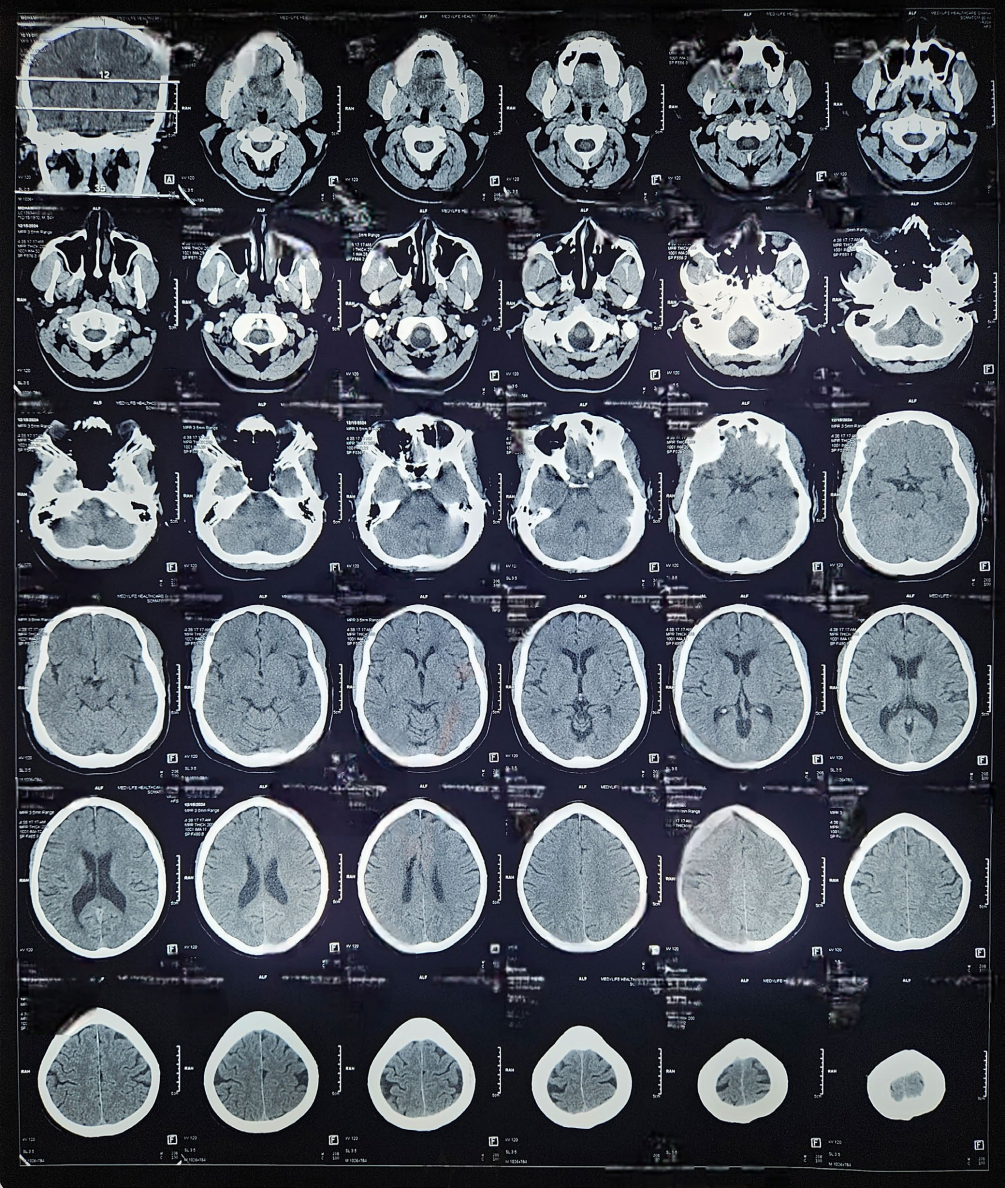
Noncontrast head CT scan showing no abnormalities.

A head CT scan requested (Figure 1) showed no abnormalities; no ischemic changes or hemorrhage, normal ventricles, and no signs of inflammation. His biochemical workup showed elevated lactate dehydrogenase (LDH) and low‐density lipoprotein (LDL) cholesterol. An ECG was done, which was normal, as well as an HIV test, which was negative. The most remarkable investigation was a positive Venereal Disease Research Laboratory (VDRL). This was followed up with a *Treponema pallidum* hemagglutination assay (TPHA), which was also positive; hence, a diagnosis of neurosyphilis was settled on. Due to the unavailability of test equipment and also to prevent a delay in management, a cerebrospinal fluid (CSF) analysis was not done. Also, with the improvement of symptoms on starting therapy, the patient requested to skip the invasive procedure. Further testing, such as MRI, was also deferred due to its inaccessibility and the significant financial constraints faced by the patient. He was started on 2.4 MIU of benzathine penicillin weekly, as well as atorvastatin for his hyperlipidemia, and the response was quick. Within 24 h of receiving his first dose, he was now oriented to time, person, and place and could engage in short conversations. His ataxic gait was also improving, as he could take multiple steps without falling. Within 48 h, he could converse and walk with minimal discomfort. He was discharged home after 5 days on admission and seen at the clinic on review within a week. His VDRL and TPHA were repeated, and remarkably, VDRL was negative, while TPHA remained positive. He continued on his penicillin shots to complete three doses. His wife was tested as well, but her tests were negative, and she had also never been symptomatic of the primary disease.

## 3 Discussion

### 3.1 Diagnostic Challenges of Neurosyphilis

Neurosyphilis remains a diagnostic challenge despite the availability of serological tests and advanced imaging techniques. The clinical presentation, as seen in this case report, can be subtle and nonspecific, with symptoms, such as incoherent speech, ataxia, and bladder incontinence mimicking several other neurological conditions like stroke, cerebellitis, normal pressure hydrocephalus, and vitamin B12 deficiency. This can delay the correct diagnosis, especially in the absence of clear prior risk factors or typical syphilitic lesions. As in this case, a thorough history and clinical examination often serve as the first clues leading to the suspicion of neurosyphilis, but diagnostic confirmation requires serological testing and CSF analysis, which are not always conclusive or readily available [4, 5]. Again, the progression of neurosyphilis is thought to be gradual, especially that involving the dorsal column (tabes dorsalis), and is usually considered a manifestation of tertiary syphilis, which takes years to develop; however, it can happen at any stage of the disease [6].

### 3.2 The Role of Serum LDH in Diagnosing Neurosyphilis

The patient's elevated LDH levels in the biochemical workup are noteworthy. LDH is a nonspecific marker of tissue damage and can be elevated in a variety of conditions, including infections, malignancies, and neurodegenerative diseases [7]. In the context of neurosyphilis, the presence of elevated LDH in the serum may reflect systemic involvement, as syphilis, especially in its tertiary stages, can cause widespread tissue damage [8]. However, its role as a diagnostic marker for neurosyphilis is not well established, and its elevation should be interpreted alongside other clinical findings and investigations [9].

### 3.3 Inconsistent CSF Analysis and Diagnostic Delays

One of the main challenges in diagnosing neurosyphilis is the reliance on CSF analysis, which may not always yield definitive results, especially in the early stages of the disease [10]. Although CSF analysis is the most widely preferred investigation for diagnosing neurosyphilis, it is not always diagnostic, as the CSF may appear normal in many cases, especially in early or mild cases. The presence of specific syphilitic antibodies in the CSF may take time to develop [9, 10, 11]. This often results in diagnostic uncertainty and delayed treatment, as clinicians may be hesitant to start empiric therapy without clear CSF findings. Since the therapy does not change much, it appears disadvantageous to delay its commencement, especially in places with limited diagnostic resources. This patient, for instance, responded within a day of treatment commencement, which is evidence of the need to avoid delaying therapy. It is important to recognize that in cases where there is a high clinical suspicion of neurosyphilis, empirical treatment with penicillin should not be delayed even in the absence of confirmatory CSF findings, as the disease can progress rapidly and result in irreversible neurological damage [4]. Unlike this patient, who almost immediately began responding positively, others may experience adverse reactions, such as the Jarisch–Herxheimer, procaine, or allergic reactions. The Jarisch–Herxheimer reaction is a transient systemic reaction to endotoxin‐like substances that are released systemically after the start of therapy and includes symptoms like fever, myalgia, headaches, hypotension, etc. [11, 12, 13] It usually resolves within 24 h. The procaine reaction results from accidental intravenous injection of procaine penicillin and is characterized by severe anxiety and fear of impending death, sometimes hallucinations and seizures [14]. Patients should be informed and counseled about the possibility of these reactions, which are manageable to ease discomfort.

When performed, however, CSF analysis in neurosyphilis typically shows an elevated white blood cell count (pleocytosis), elevated protein levels, and a positive VDRL test [15].

### 3.4 Serological Testing: VDRL and TPHA

VDRL is a nontreponemal test that detects antibodies to cardiolipin, which are produced in response to infection with *Treponema pallidum* [16]. While the VDRL test is useful for screening, it is less specific and can yield false‐positive results in conditions, such as autoimmune diseases, viral infections, or pregnancy [17]. The TPHA is a treponemal test that detects antibodies specific to *Treponema pallidum* and is more specific than VDRL. In this case, the positive VDRL and TPHA, along with the clinical presentation, led to a diagnosis of neurosyphilis.

Interestingly, while VDRL became negative after treatment, the TPHA remained positive, which is typical in syphilis infections. The VDRL can revert to negative after effective treatment, reflecting the resolution of the active infection, while the TPHA remains positive for years, as it detects antibodies that persist even after successful treatment [17, 18, 19]. This discrepancy highlights the importance of using both tests in diagnosing syphilis and monitoring treatment response. The patient's response to penicillin therapy was dramatic, with rapid improvement in symptoms within 24–48 h, further supporting the diagnosis of neurosyphilis and the effectiveness of early treatment.

## 4 Conclusion

Neurosyphilis presents significant diagnostic challenges due to its variable clinical manifestations and the inconsistency of CSF findings, which can delay treatment initiation. In this case, the clinical presentation, along with positive serological tests, led to a diagnosis of neurosyphilis despite the absence of confirmatory CSF analysis. The elevated serum LDH, while suggestive of systemic involvement, did not provide definitive diagnostic information. Ultimately, early empirical treatment with penicillin resulted in a rapid clinical improvement, underscoring the importance of prompt recognition and intervention. Clinicians should maintain a high index of suspicion for neurosyphilis, particularly in patients with risk factors for syphilis, and should not delay treatment even when CSF results are inconclusive.

## Author Contributions

**Samuel Amo‐Tachie:** conceptualization, data curation, formal analysis, investigation, methodology, resources, validation, visualization, writing – original draft, writing – review and editing.

## Consent

A written informed consent was obtained from the patient to publish this report in accordance with the journal's patient consent policy.

## Conflicts of Interest

The author declares no conflicts of interest.

## Data Availability

The data that support the findings of this study are available on request from the corresponding author [S.A.‐T.].
